# Do handgrip strength and dexterity predict respiratory function in neuromuscular disease?

**DOI:** 10.1055/s-0042-1758757

**Published:** 2022-12-28

**Authors:** Ertugrul Safran, Aysel Yildiz Ozer, Hulya Nilgun Gurses

**Affiliations:** 1 Bezmialem Vakif University, Faculty of Health Sciences, Division of Physiotherapy and Rehabilitation, Istanbul, Turkey.; 2 Marmara University, Faculty of Health Sciences, Division of Physiotherapy and Rehabilitation, Department of Cardiopulmonary Physiotherapy Rehabilitation, Istanbul, Turkey.; 3 Bezmialem Vakif University, Faculty of Health Sciences, Division of Physiotherapy and Rehabilitation, Department of Cardiopulmonary Physiotherapy and Rehabilitation, Istanbul, Turkey.

**Keywords:** Neuromuscular Diseases, Motor Skills, Respiratory Function Tests, Hand Strength, Doenças Neuromusculares, Destreza Motora, Testes de Função Respiratória, Força da Mão

## Abstract

**Background** Neuromuscular diseases are acquired or inherited diseases that affect the function of the muscles in our body, including respiratory muscles.

**Objective** We aimed to discover more cost-effective and practical tools to predict respiratory function status, which causes serious problems with patients with neuromuscular disease.

**Methods** The Vignos and Brooke Upper Extremity Functional Scales were used to evaluate functional status for patient recruitment. The handgrip strength and dexterity of patients were measured using a dynamometer and nine-hole peg test. Respiratory function parameters: forced vital capacity, forced expiratory volume in one second, and peak expiratory flow were evaluated using spirometry.

**Results** The mean age of the 30 patients was 11.5 ± 3.79 years old. Significant relationships were found between nine-hole-peg-test scores and respiratory function parameters on both sides. Significant correlations were found between both handgrip strength and respiratory function parameters ( *p* < 0.05). In the linear regression analysis, it was seen that the forced expiratory volume in 1 second, and peak expiratory flow values could be explained in different percentages ( *p* < 0.05).

**Conclusions** Handgrip strength and dexterity measurements can be used as indicators for estimating respiratory function parameters in terms of cost and accessibility, although it is known that they will not replace respiratory function tests.

## Resumo

**Antecedentes** As doenças neuromusculares são doenças adquiridas ou hereditárias que afetam a função dos músculos do nosso corpo, incluindo os músculos respiratórios.

**Objetivo** Nosso objetivo foi descobrir ferramentas mais práticas e econômicas para prever o estado da função respiratória, que causa sérios problemas em pacientes com doença neuromuscular.

**Métodos** As Escalas Funcionais da Extremidade Superior de Vignos e Brooke foram utilizadas para avaliar o estado funcional para recrutamento de pacientes. A força de preensão manual e a destreza dos pacientes foram medidas por meio de um dinamômetro e do teste de nove buracos. Os parâmetros da função respiratória: capacidade vital forçada, volume expiratório forçado no primeiro segundo e pico de fluxo expiratório foram avaliados por meio da espirometria.

**Resultados** A média de idade dos 30 pacientes foi de 11,5 ± 3,79 anos. Relações significativas foram encontradas entre as pontuações do teste de nove buracos e os parâmetros da função respiratória em ambos os lados. Correlações significativas foram encontradas entre a força de preensão manual e os parâmetros da função respiratória ( *p* < 0,05). Na análise de regressão linear, observou-se que o volume expiratório forçado no primeiro segundo e os valores de pico de fluxo expiratório puderam ser explicados em diferentes percentuais ( *p* < 0,05).

**Conclusões** As medidas de força de preensão manual e destreza podem ser utilizadas como indicadores para estimar parâmetros da função respiratória em termos de custo e acessibilidade, embora se saiba que não substituirão os testes de função respiratória.

## INTRODUCTION

Neuromuscular diseases (NMDs) are acquired or hereditary diseases of the spinal anterior horn cells, peripheral nerves, neuromuscular junction, and muscles. 1 Neuromuscular disease-induced insufficiencies are predisposing factors caused by multisegmental disorders in addition to the musculoskeletal system. Respiratory system involvement affects many patients with these problems in different periods of life. Respiratory muscle strength loss causes ineffective cough and hypoventilation. Although this condition is dormant at the beginning, it paves the way for atelectasis, pneumonia, and respiratory failure, which may also be observed during later stages when the muscle strength is weaker. Dysfunction due to the weakness in respiratory muscles is one of the major problems that cause mortality and morbidity in patients with NMDs of different ages. Therefore, the follow-up of respiratory functions in patients with progressive NMD is recommended. 1 2 3

Many practitioners and researchers are trying to work on increasing the life span and/or quality of life with medical and rehabilitative approaches because NMDs have a crucial life-threatening natural progression. Before choosing these approaches, first, it is necessary to determine the current condition of the patient. Fatigue due to the disease is the most important limitation that causes the comprehensive evaluation periods to be kept short. At this point, using simple clinical assessment tools is useful for physicians and patients in overcoming time limitations and fatigue risk in practice. Today, functional outcome measures and respiratory assessments are the most common methods for the determination of the condition of patients.

Handgrip strength measurement is a widely used peripheral muscle strength assessment method in healthy subjects or patients. It is also known that many functional parameters interact with grip strength. 4 5 Similarly, hand dexterity assessment is a useful tool to evaluate many functional parameters such as cognitive and functional performance due to its cost-effective and practical nature. 6 7 Although spirometry and respiratory muscle strength measurements are used to assess respiratory function, these may not always be available in pediatric/adult rehabilitation centers due to their elevated cost and accessibility. In addition, respiratory muscle strength loss and fatigue factors that develop due to the nature of NMD may negatively affect the reliability of the results.

Practitioners working with patients with NMD should be aware of the effects of muscle weakness and cognitive dysfunction on the respiratory system. Symptoms can be insidious and may cause progressive loss of function, respiratory failure, and even death. 8 Although the effects of grip strength and cognitive functions on respiratory function have been shown in different diseases, 9 10 there is a dearth of literature in NMD. Many studies have focused on the relationship between the ambulation levels of patients and respiratory functions and upper extremity functional status. However, the relationship between upper extremity functions and respiratory functions in patients with NMD remains unclear.

Practical and predictive approaches are needed for the evaluation of the respiratory function, since it is one of the most important monitoring parameters in individuals with NMD and the accessibility of the pulmonary function test is not always possible (such as during the pandemic period). Furthermore, it is relatively expensive as the pulmonary function test must be performed by a specialist using a medical device. Besides, it may be difficult to complete the test in small children and doubtful results may be obtained in this group. For these reasons, although it is not intended to replace the pulmonary function test, there is a clinical need to examine the novel parameters and question their usability in predicting pulmonary function losses nowadays. Considering the relationship between upper extremity functions and respiratory functions in NMD, we think that it can be helpful in predicting respiratory functions, especially the involvement of accessory respiratory muscles.

In the present study, we aimed to investigate the relationship of handgrip strength and dexterity with respiratory functions, and to find out whether these measurements were more accessible and practical tools to predict respiratory functional status in patients with NMD.

## METHODS

Written informed consent was received from the families of the children and from patients ≥18 years old for participating in the study. The research was approved by the Medipol University Ethics Committee, and it was conducted in accordance with the Helsinki Declaration. The study was registered in the International Clinical Trials Registry ( www.clinicaltrials.gov ; ClinicalTrials.gov Identifier: NCTXXXXXX).

### Participants

Thirty patients with NMD aged between 6 and 18 years old who were referred by a Counseling and Research Center to a rehabilitation center were included in the study. Patients who met the following criteria were included in the study: being diagnosed as having NMD, a Vignos scale of stage 5 or below (walking without assistance), and a Brooke scale of stage 1 to 4. Patients with severe systemic disease, ambulatory problems, cognitive problems, and upper extremity deformities were excluded.

### Assessments

Upper Extremity Functional Level and Ambulation Status: The most used functional scales for determining the severity of disease and for grading disease are the Brooke and Vignos scales. Both scales were designed primarily for patients with Duchenne muscular dystrophy (DMD) and are currently used for many NMDs. 11 The upper extremity functional levels of the patients included in our study were determined using the 6-point Brooke Upper Extremity Functional Scale (BUEFS). 12 To determine the patients to be included in the study, the ambulation levels of the patients were evaluated using the Vignos scale. Vignos is a 10-point scale used to evaluate lower extremity functions and the ambulation status of patients with NMDs. 13 Patients who had a Vignos scale ≤ stage 5 (walking without assistance) and a Brooke scale of stage 1 to 4 were included in the study.

#### *Handgrip strength*

A Jamar (Lafayette Instrument, Lafayette-IN, USA) handgrip dynamometer, which was recommended by the American Society of Hand Therapists, was used in the measurement of the handgrip strength of the patients. Jamar dynamometers had high validity and reliability in many studies and therefore were accepted as the gold standard for this assessment. 14 Participants were instructed to sit in a straight-backed chair with their feet placed flat on the floor, shoulder in abduction and neutral rotation, elbow flexion at 90°, with the forearm and wrist in the neutral position. The participants were asked to squeeze the dynamometer with as much force as possible and to perform maximal isometric grip. A total of three measurements were made and a 1-minute rest period was given after each measurement. The average of the three measurements was recorded. 15

#### *Hand dexterity*

The nine-hole peg test (NHPT) was used to measure hand dexterity. 16 Patients were asked to take nine wood sticks, one by one, from the container, place them into holes, then collect them from the holes and put them back in the container. In this test procedure, the board should be positioned in the patient's midline. The hand that is not evaluated should hold the edge of the board and provide stability. The times to complete the test were recorded using a stopwatch in seconds. 17 A total of three measurements were made and a 1-minute rest period was given after each measurement. An average of the three measurements was recorded. 18

#### *Assessment of respiratory function parameters*

Respiratory function parameters were evaluated using a spirometer (COSMED Pony FX; COSMED, Italy). The measurements were taken in accordance with the standardization criteria jointly determined by the American Thoracic Society (ATS) and the European Respiratory Society (ERS). 19 20 In the study, the forced expiratory volume in one second (FEV _1_ ), forced vital capacity (FVC), FEV _1_ /FVC, peak expiratory flow (PEF), and predictive values of these parameters were examined.

### Statistical analysis

IBM SPSS Statistics for Windows version 20 (IBM Corp., Armonk. NY, USA) was used for data analysis. All data were analyzed using the Kolmogorov-Smirnov test to determine distribution characteristics. The relationship between clinical parameters was evaluated according to the distribution characteristics of the data using Pearson or Spearman correlation coefficient analysis. Linear regression analysis was conducted to analyze independent predictors of respiratory function parameters. Statistical significance was accepted as *p* < 0.05 in all analyses.

## RESULTS

**Figure 1.**
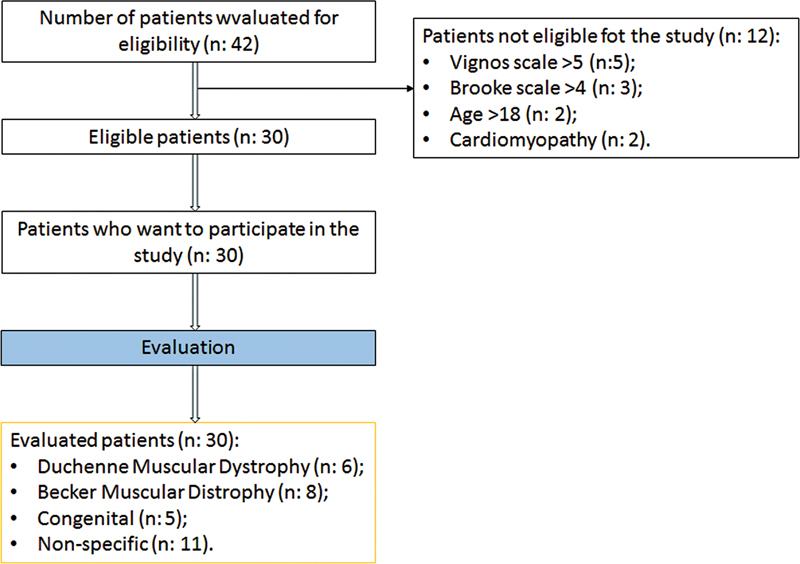
Flow chart of the study.

Forty-two patients were evaluated within the scope of the study. Twelve patients were excluded according to our inclusion-exclusion criteria. Thirty patients with NMD, 14 females and 16 males, between the ages of 6 and 18 (mean: 11.5 ± 3.79) years old who met the criteria were included in the study ( Figure 1 ). The dominant hand was the right hand in all patients. Half of the patients ( *n* = 15) had a family history. Nonspecific myopathy ( *n* = 11) was the most frequent NMD type observed in these patients. The characteristics of the participants are shown in Table 1 .

**Table 1:** Characteristics of the patients

		Mean ± SD
Age (years old)		11.5 ± 3.78
Height (cm)		133.9 ± 33.06
Weight (kg)		39.03 ± 18.41
Vignos stage ( *n* )	I	3
	II	7
	III	11
	IV	5
	V	4
Brooke stage ( *n* )	I	10
	II	7
	III	10
	IV	3
Type of neuromuscular disease		*n*
Duchenne muscular dystrophy		6
Becker muscular dystrophy		8
Congenital		5
Nonspecific		11

Abbreviations: m, mean; n, number; SD, standard deviation.

The grip strength and NHPT scores of the patients were better on the right side ( *p* < 0.05); however, the handgrip strength of many of patients (for dominant side/non-dominant side: 33.33/50% patients) and dexterity measurements (for dominant and non-dominant sides in 86.67% patients) were below the normative values. In addition, although ambulated patients were included in our study, the pulmonary functions of a significant number of patients were < 80% (FEV1%, FVC% and PEF% predicted; *n* = 18; *n* = 19 and *n* = 21 patients, respectively) ( Table 2 ).

**Table 2:** Results of handgrip strength, dexterity, and pulmonary function measurements of patients with neuromuscular diseases

Parameter		Mean ± SD
Handgrip strength (kgf)	Right side	7.74 ± 4.46
	Left side	6.60 ± 4.30
	*p-value*	< 0.001
Nine-hole peg test (sec)	Right side	30.99 ± 2.33
	Left side	32.84 ± 14
	*p-value*	0.002
FEV _1_ liter		1.54 ± 0.67
FEV _1%_ (pred)		73 ± 17.71
FVC liter		1.74 ± 0.77
FVC % (pred)		76.30 ± 17.90
FEV _1_ / FVC		91.19 ± 14.69
PEF liter		2.58 ± 1.25
PEF % (pred)		64.53 ± 20.57

Abbreviations: FEV _1_ , Forced Expiratory Volume in one second; FVC, Forced Vital Capacity; PEF, Peak Expiratory Flow; kgf, Kilogram-force; sd: standard deviation; sec, Seconds; p, Comparison between right and left side; pred, Predicted value.

Significant negative correlations were found between the NHPT scores of both sides and the percentage predicted values of FEV _1_ and PEF, and PEF (L) ( *p* < 0.05). There were also significant correlations between the right and left handgrip strength of the participants and the percentage predicted values of FEV _1_ and PEF, and PEF (L) ( *p* < 0.05). Statistical analysis showing the relationship between all upper extremity parameters and respiratory function test parameters are shown in Table 3 .

**Table 3:** Relationship between upper extremity functions and pulmonary function

	FEV _1_ L	FEV _1_ % (pred)	FVC L	FVC % (pred)	PEF L	PEF % (pred)
**Right NHPT**	r _s_ = - 0.272 *p = * 0.146	r _s _ = -0.474 *p = * 0.008	r _s _ = - 0.264 *p = * 0.146	r _s_ = - 0.160 *p = * 0.398	r _s _ = - 0.568 *p = * 0.001	r _s _ = - 0.737 *p = * 0.001
**Left NHPT**	r _s _ = - 0.234 *p = * 0.213	r _s _ = - 0.484 *p = * 0.007	r _s _ = - 0.226 *p = * 0.230	r _s_ = - 0.204 *p = * 0.280	r _s _ = - 0.550 *p = * 0.002	r _s _ = - 0.732 *p* < 0.001
**Right HGS**	r _p _ = 0.580 *p = * 0.001	r _p _ = 0.142 *p = * 0.453	r _p _ = 0.427 *p = * 0.02	r _p _ = 0.121 *p = * 0.525	r _p _ = .740 *p* < 0.001	r _p _ = 0.390 *p* = 0.033
**Left HGS**	r _p _ = 0.594 *p* = 0.001	r _p _ = 0.358 *p = * 0.174	r _p _ = 0.465 *p = * 0.01	r _p _ = 0.079 *p = * 0.676	r _p _ = .766 *p* < 0.001	r _p _ = 0.423 *p = * 0.02

Abbreviations: FEV _1_ , Forced Expiratory Volume in one second; FVC, Forced Vital Capacity; HGS, Handgrip strength; L, Liter; NHPT, Nine-hole Peg Test; PEF, Peak Expiratory Flow; p, Significance; pred, Predicted value; r _s_ , Spearman's correlation coefficient; r _p_ , Pearson correlation coefficient.

We examined the handgrip strength to predict respiratory function parameters using linear regression analysis and found that the right-handgrip strength value could explain 33.6, 54.8, and 15.2% of the variances of FEV _1_ (L), PEF (L), and PEF (% predicted) values, respectively. The left-handgrip strength value could explain 35.2, 58.6, and 17.9% of the variances in the FEV _1_ (L), PEF (L), and PEF (% predicted) values, respectively. Concerning the level of ability of the hand dexterity scores, we found that the right NHPT score could explain 17.4, 31.6, and 57% of the variances in the FEV _1_ (% predicted) PEF (L) and PEF (% predicted) values, respectively, and the left hand NHPT score could explain 17, 53.9, and 27.6% of the variances in the FEV _1_ (% predicted), PEF (% predicted) and PEF (L) values, respectively ( Table 4 ).

**Table 4:** Regression analysis of respiratory function parameters

Dependent Variable	Predictors	R	R ^2^	p
**FEV 1 liter**	Right handgrip strength	0.580	0.336	**0.001**
	Left handgrip strength	0.594	0.352	**0.001**
	Right nine-hole peg test	0.266	0.071	0.155
	Left nine-hole peg test	0.229	0.053	0.223
**FEV1% (pred)**	Right handgrip strength	0.142	0.020	0.956
	Left handgrip strength	0.174	0.030	0.358
	Right nine-hole peg test	0.417	0.174	**0.022**
	Left nine-hole peg test	0.413	0.170	**0.023**
**PEF liter**	Right handgrip strength	0.740	0.548	**< 0.001**
	Left handgrip strength	0.766	0.586	**< 0.001**
	Right nine-hole peg test	0.562	0.316	**0.001**
	Left nine-hole peg test	0.525	0.276	**0.003**
**PEF % (pred)**	Right handgrip strength	0.390	0.152	**0.033**
	Left handgrip strength	0.423	0.179	**0.020**
	Right nine-hole peg test	0.755	0.570	**< 0.001**
	Left nine-hole peg test	0.734	0.539	**< 0.001**

Abbreviations: FEV _1_ , Forced Expiratory Volume in one second; FVC, Forced Vital Capacity; PEF, Peak Expiratory Flow; p, Significance; pred, Predicted value; R, Coefficient of regression; R ^2^ , Coefficient of determination; Note: *Bold numbers indicate statistically significant.

## DISCUSSION

In the present study, handgrip strength and dexterity and their relationship with respiratory function in patients with NMD were investigated. In this context, the study also investigated whether they were more accessible and practical tools to predict respiratory function status in these patients.

Our results showed that there were significant relationships among handgrip strength, dexterity, and pulmonary function. According to the regression analysis, we found that bilateral grip strengths could explain the variances of FEV _1_ (L), PEF (L) and PEF (% predicted) values. Especially the fact that the right and left side grip strength tests explain the PEF (L) value at the level of 54.8 and 58.6%, respectively, is a significant prediction. Also, we found that hand dexterities could explain the variances of FEV _1_ (% predicted), PEF (L) and PEF (% predicted) values according to our regression analysis. When we look at our results related to manual dexterity, we observe positive data in terms of usability of manual dexterity scores measured with the nine-hole peg test, which is frequently used for performance and cognitive evaluations, as an auxiliary equipment in predicting respiratory functions in individuals with NMD. The fact that the right- and left-hand dexterity scores explain the PEF (% predicted) value by 57 and 54%, respectively, is a significant ratio in terms of being used as a predictor. These results confirm our hypothesis that low hand grip strength and dexterity indicate poor respiratory function. Hand grip strength and dexterity scores are possible practical tools that can be used to predict pulmonary function test scores.

The patients with NMD who were included in previous studies that evaluated upper extremity functions were mostly nonambulatory. 21 22 However, a continued/protected ambulation level/skill is important for studies that evaluate upper extremity functions and pulmonary status in terms of research reliability. We evaluated patients who had mobility, and nonambulatory patients were excluded from the study because of the possibility that ambulation levels might affect respiratory functions and test results.

Mercuri et al. 23 emphasized the validity of the Brooke scale for upper extremity assessment. Recently, Davoli et al. 24 also reported that The Brooke Upper Extremity Instrument was one of the most used tools. Furthermore, the Brooke and Vignos scales showed a positive relationship between functional ability and FVC and total lung capacity. 25 26 Another scale suggested for advanced stage NMD, the Egen Classification Scale, which includes questions about respiratory dysfunction (coughing, talking, and wellbeing), also confirmed the relationship between worse respiratory parameters and functional level. 27

Several neuroscience studies in the literature examined the relationship between upper extremity functions and respiratory functions. 28 29 Lee et al. 28 demonstrated a strong correlation between upper extremity functions and pulmonary function test measurements. Ricotti et al. 29 examined pulmonary, upper-extremity function, and dynamometry results in ambulatory and non-ambulatory patients with DMD. Jeong et al. 30 showed that low grip strength was a risk factor in respiratory diseases such as chronic obstructive pulmonary disease. In addition, recently, Zhu et al. 31 stated that grip strength measurements might be an indicator to monitor the cardiopulmonary disease process. On the other hand, Mutluay et al. 32 reported that upper extremity strengthening programs positively affected respiratory parameters in patients with multiple sclerosis. They also said that strengthening the accessory respiratory muscles of the trunk and upper extremities would increase respiratory functions. In the present study, we found that hand grip strength values had positive moderate correlations with FEV _1_ (L), FVC (L), and PEF (% predicted), while having strong correlations with PEF (L) values. Our findings were compatible with the literature. Respiratory care interventions for complications caused by respiratory muscles impairment and preventive pulmonary approaches are important for sustaining functions and quality of life in patients with NMD. Thus, there is a need for simple assessment tools that can indicate respiratory problems at the early stages of the disease, when the patients are still ambulatory. Unfortunately, studies in the literature are still insufficient in this regard.

Handgrip strength measurements can be used on their own instead of global muscle strength measurements, along with different parameters. Many studies have also been conducted in healthy individuals and it has been stated that handgrip strength measurement can be used to predict respiratory function values. 5 33 Considering their relationship between handgrip and respiratory functions, it was seen that individuals with greater handgrip strength had better respiratory function values. 34 It has been shown in studies that grip strength is decreased in patients with NMD compared with healthy individuals, and similar to our study, the dominant handgrip strength is slightly higher than the no-dominant hand. 35 36 Furthermore, weakness in the proximal shoulder girdle in the early stages continues towards the elbows, wrist and hand muscles as the disease progresses in this group. 37

Recently, Ricotti et al. 29 focused on outcome measures in DMD that were independent of ambulation and highlighted across the ambulatory stages of the disease according to its progression. They measured distal upper limb strength using MyoGrip and MyoPinch dynamometers and used Performance of the Upper Limb (PUL) and respiratory function. They found a low decrease over a year for the shoulder subdomain. This result was in line with the proximal to the distal progression of the disease. Annual grip values also decreased more rapidly in ambulant patients. However, they found no significant change in FVC (% predicted) but they determined a decline in PEF (% predicted), which was seen both in the ambulant stage of the disease and further into the nonambulant stage with an annual deterioration of PEF (% predicted). This decreased PEF (% predicted) value was attributed to the already impaired maximum expiratory muscle pressure. The handgrip strength, dexterity measurements, and respiratory functions of our patients showed different degrees of decrease than normative values, similar to the results of Ricotti et al. 29 In our evaluations, distal grip strength and dexterity measurements of both sides were found to be weaker than normal values in a significant number of patients. When the results of our study were examined in terms of handgrip and dexterity, it was seen that our patients had lower mean values compared with healthy individuals in the same age group. 36 Additionally, it is interesting that the results of grasping and dexterity were found to be related to PEF (% predicted) and PEF liters in our study. It may be considered that these measurements should be made from the early stages of the disease in patients with NMD in terms of determining risks that may be indicated by low grip strength because it is known that pulmonary functions worsen at later stages in patients with NMD.

One of the important problems reported in patients with NMD is fatigue. 38 With the progression of the disease, fatigue becomes evident earlier and in short-term activities that require low effort. In addition, Kaminska et al. 39 reported that it is difficult to maintain an effective mouth seal or to sustain a maximal inspiratory effort in children, and that there is a possibility of obtaining misleadingly low values in respiratory muscle strength measurements. We think that in addition to progressive fatigue, patient-related factors in practice may reduce the reliability of the data obtained from pulmonary function tests. Handgrip is a quick and easy measurement for both ambulatory and nonambulatory NMD patients. In the present study, we used upper extremity handgrip strength and dexterity measurements, which are simple and easy assessment tools. We found that the handgrip tests showed a moderate positive correlation with FEV _1_ (L), a low positive correlation with FVC (L) and PEF (% predicted), and a strong positive correlation with PEF (L). In addition, dexterity test results showed negative correlations with FEV _1_ (% predicted), PEF (% predicted) and PEF (L) values. Our results are important because they indicate the deterioration in the respiratory functions of the patients in the early period. Although our limited number of cases did not allow advanced statistical analyses, the results of the regression analyses also supported our findings. In the light of our findings, monitoring the handgrip measurements from the early stages may help health professionals in terms of clinical evaluation in these children.

The limited number of participants seems to be a limitation and the small sample size precluded advanced subanalyses. Our patient population consisted of the referral pattern to our center and not all patients of the same age could not be referred. However, when the literature and the patient portfolio in the clinic are examined, it would be difficult to include high numbers of patients in the study group, so our number of patients can be considered as reasonable. More importantly, the present data show the natural history of patients with ambulatory NMD managed without respiratory support and, therefore, our results are valuable.

Our study is important in terms of filling a gap in the literature; significant positive correlations and negative correlations were found between handgrip strength and hand dexterity with respiratory function parameters, respectively. The results showed that handgrip strength and dexterity measurement follow-ups in patients with NMD can be used as practical tools to predict respiratory function in situations in which respiratory function tests are not available due to lack of facilities and high cost.

In conclusion, although we do not claim to replace common respiratory function tests, it has been shown that handgrip strength and dexterity measurements may predict respiratory function parameters. Although we consider the nonperformance of respiratory muscle strength measurements in addition to pulmonary function tests as a limitation, we can point to the lack of this test in many clinics as a justification, in terms of cost. Future studies may also investigate the results from that perspective by making comparisons with healthy participants.

## References

[1] Kennedy J D Martin A J Chronic respiratory failure and neuromuscular disease Pediatr Clin North Am 2009 56 01 261 273, xii 10.1016/j.pcl.2008.10.011 19135591

[2] Perrin C Unterborn J N Ambrosio C D Hill N S Pulmonary complications of chronic neuromuscular diseases and their management Muscle Nerve 2004 29 01 5 27 10.1002/mus.10487 14694494

[3] Allen J Respiratory function in children with neuromuscular disease Monaldi Archives for Chest Disease/Arch Monaldi Mal Torace 1996 51 03 230 235PubMed 8766200

[4] Seferian A M Moraux A Annoussamy M Upper limb strength and function changes during a one-year follow-up in non-ambulant patients with Duchenne Muscular Dystrophy: an observational multicenter trial PLoS One 2015 10 02 e0113999 10.1371/journal.pone.0113999 25643053 PMC4314080

[5] Han C H Chung J H Association between hand grip strength and spirometric parameters: Korean National health and Nutrition Examination Survey (KNHANES) J Thorac Dis 2018 10 11 6002 6009 10.21037/jtd.2018.10.09 30622771 PMC6297404

[6] Yozbatiran N Baskurt F Baskurt Z Ozakbas S Idiman E Motor assessment of upper extremity function and its relation with fatigue, cognitive function and quality of life in multiple sclerosis patients J Neurol Sci 2006 246(1-2):117 122 10.1016/j.jns.2006.02.018 16678208

[7] Niu H-X Wang R-H Xu H-L Nine-hole peg test and ten-meter walk test for evaluating functional loss in Chinese charcot-marie-tooth disease Chin Med J (Engl) 2017 130 15 1773 1778 10.4103/0366-6999.211550 28748848 PMC5547827

[8] Panitch H B Respiratory implications of pediatric neuromuscular disease Respir Care 2017 62 06 826 848 10.4187/respcare.05250 28546380

[9] Chyou P-H White L R Yano K Pulmonary function measures as predictors and correlates of cognitive functioning in later life Am J Epidemiol 1996 143 08 750 756 10.1093/oxfordjournals.aje.a008812 8610684

[10] Richards M Strachan D Hardy R Kuh D Wadsworth M Lung function and cognitive ability in a longitudinal birth cohort study Psychosom Med 2005 67 04 602 608 10.1097/01.psy.0000170337.51848.68 16046374

[11] Lu Y-M Lue Y-J Strength and functional measurement for patients with muscular dystrophy Muscular Dystrophy 2012 32 03 671 688

[12] Brooke M H Griggs R C Mendell J R Fenichel G M Shumate J B Pellegrino R J Clinical trial in Duchenne dystrophy. I. The design of the protocol Muscle Nerve 1981 4 03 186 197 10.1002/mus.880040304 7017401

[13] Vignos P J Jr Archibald K C Maintenance of ambulation in childhood muscular dystrophy J Chronic Dis 1960 12 02 273 290 10.1016/0021-9681(60)90105-3 13842210

[14] Bellace J V Healy D Besser M P Byron T Hohman L Validity of the Dexter Evaluation System's Jamar dynamometer attachment for assessment of hand grip strength in a normal population J Hand Ther 2000 13 01 46 51 10.1016/S0894-1130(00)80052-6 10718222

[15] Roberts H C Denison H J Martin H J A review of the measurement of grip strength in clinical and epidemiological studies: towards a standardised approach Age Ageing 2011 40 04 423 429 10.1093/ageing/afr051 21624928

[16] Wang Y-C Bohannon R W Kapellusch J Garg A Gershon R C Dexterity as measured with the 9-Hole Peg Test (9-HPT) across the age span J Hand Ther 2015 28 01 53 59, quiz 60 10.1016/j.jht.2014.09.002 25449717

[17] Smith Y A Hong E Presson C Normative and validation studies of the Nine-hole Peg Test with children Percept Mot Skills 2000 90(3 Pt 1):823 843 10.2466/pms.2000.90.3.823 10883762

[18] Haidar S G Kumar D Bassi R S Deshmukh S C Average versus maximum grip strength: which is more consistent? J Hand Surg Br 2004 29 01 82 84 10.1016/j.jhsb.2003.09.012 14734079

[19] Graham B L Steenbruggen I Miller M R Standardization of spirometry 2019 update. An official American thoracic society and European respiratory society technical statement Am J Respir Crit Care Med 2019 200 08 e70 e88 10.1164/rccm.201908-1590ST 31613151 PMC6794117

[20] Wanger J Clausen J L Coates A Standardisation of the measurement of lung volumes Eur Respir J 2005 26 03 511 522 10.1183/09031936.05.00035005 16135736

[21] Servais L Canal A De Coninck N Upper limb evaluation in non-ambulatory patients with neuromuscular disorders Neuromuscul Disord 2010 20(9–10):669 10.1016/j.nmd.2010.07.232

[22] MDA DMD Clinical Research Network Connolly A M Malkus E C Mendell J R Outcome reliability in non-ambulatory boys/men with Duchenne muscular dystrophy Muscle Nerve 2015 51 04 522 532 10.1002/mus.24346 25056178 PMC4305351

[23] Mercuri E McDonald C Mayhew A International workshop on assessment of upper limb function in Duchenne Muscular Dystrophy: Rome, 15-16 February 2012 Neuromuscul Disord 2012 22 11 1025 1028 10.1016/j.nmd.2012.06.006 22795657 PMC3500683

[24] Davoli G BQ Cardoso J Silva G C Moreira R FC Mattiello-Sverzut A C Instruments to assess upper-limb function in children and adolescents with neuromuscular diseases: a systematic review Dev Med Child Neurol2021 Sep;63 09 1030 1037 10.1111/dmcn.14887 33834485

[25] Hapke E J Meek J C Jacobs J Pulmonary function in progressive muscular dystrophy Chest 1972 61 01 41 47 10.1378/chest.61.1.41 5049500

[26] Inkley S R Oldenburg F C Vignos P J Jr Pulmonary function in Duchenne muscular dystrophy related to stage of disease Am J Med 1974 56 03 297 306 10.1016/0002-9343(74)90611-1 4813648

[27] Brunherotti M A Sobreira C Rodrigues-Júnior A L de Assis M R Terra Filho J Baddini Martinez J A Correlations of Egen Klassifikation and Barthel Index scores with pulmonary function parameters in Duchenne muscular dystrophy Heart Lung 2007 36 02 132 139 10.1016/j.hrtlng.2006.07.006 17362794

[28] Lee H N Sawnani H Horn P S Rybalsky I Relucio L Wong B L The Performance of the Upper Limb scores correlate with pulmonary function test measures and Egen Klassifikation scores in Duchenne muscular dystrophy Neuromuscul Disord 2016 26(4-5):264 271 10.1016/j.nmd.2016.02.015 27056113

[29] Ricotti V Selby V Ridout D Respiratory and upper limb function as outcome measures in ambulant and non-ambulant subjects with Duchenne muscular dystrophy: A prospective multicentre study Neuromuscul Disord 2019 29 04 261 268 10.1016/j.nmd.2019.02.002 30852071

[30] Jeong M Kang H K Song P Hand grip strength in patients with chronic obstructive pulmonary disease Int J Chron Obstruct Pulmon Dis 2017 12 2385 2390 10.2147/COPD.S140915 28848339 PMC5557109

[31] Zhu R Li W Xia L Hand grip strength is associated with cardiopulmonary function in Chinese adults: Results from a cross-sectional study J Exerc Sci Fit 2020 18 02 57 61 10.1016/j.jesf.2019.12.001 31889964 PMC6933200

[32] Mutluay F K Demir R Ozyilmaz S Caglar A T Altintas A Gurses H N Breathing-enhanced upper extremity exercises for patients with multiple sclerosis Clin Rehabil 2007 21 07 595 602 10.1177/0269215507075492 17702701

[33] Mgbemena N C Aweto H A Tella B A Emeto T I Malau-Aduli B S Prediction of lung function using handgrip strength in healthy young adults Physiol Rep 2019 7 01 e13960 10.14814/phy2.13960 30632320 PMC6328910

[34] Chen L Liu X Wang Q Better pulmonary function is associated with greater handgrip strength in a healthy Chinese Han population BMC Pulm Med 2020 20 01 1 8 10.1186/s12890-020-1155-5 32349735 PMC7191764

[35] Mattar F L Sobreira C Hand weakness in Duchenne muscular dystrophy and its relation to physical disability Neuromuscul Disord 2008 18 03 193 198 10.1016/j.nmd.2007.11.004 18207403

[36] Häger-Ross C Rösblad B Norms for grip strength in children aged 4-16 years Acta Paediatr 2002 91 06 617 625 10.1111/j.1651-2227.2002.tb03290.x 12162590

[37] Kurillo G Han J J Abresch R T Nicorici A Yan P Bajcsy R Development and application of stereo camera-based upper extremity workspace evaluation in patients with neuromuscular diseases PLoS One 2012 7 09 e45341 10.1371/journal.pone.0045341 23028947 PMC3444448

[38] Pangalila R F van den Bos G A Bartels B Bergen M Stam H J Roebroeck M E Prevalence of fatigue, pain, and affective disorders in adults with duchenne muscular dystrophy and their associations with quality of life Arch Phys Med Rehabil 2015 96 07 1242 1247 10.1016/j.apmr.2015.02.012 25731937

[39] Kaminska M Noel F Petrof B J Optimal method for assessment of respiratory muscle strength in neuromuscular disorders using sniff nasal inspiratory pressure (SNIP) Zissel G, editor. PLoS One. 2017May;12 05 e0177723 https://doi.org/10.1371/journal.pone.0177723 10.1371/journal.pone.0177723 PMC5433762 28520769

[40] Gotthelf M Townsend D Durfee W A video game based hand grip system for measuring muscle force in children J Neuroeng Rehabil 2021 18 01 113 10.1186/s12984-021-00908-1 34246310 PMC8272373

